# Inclusion of Cultural and Linguistic Diversity in COVID-19 Public Health Research: Research Design Adaptations to Seek Different Perspectives in Victoria, Australia

**DOI:** 10.3390/ijerph20032320

**Published:** 2023-01-28

**Authors:** Lisa Gibbs, Alexander J. Thomas, Alison Coelho, Adil Al-Qassas, Karen Block, Niamh Meagher, Limya Eisa, Stephanie Fletcher-Lartey, Tianhui Ke, Phoebe Kerr, Edwin Jit Leung Kwong, Colin MacDougall, Deng Malith, Katitza Marinkovic Chavez, Deborah Osborne, David J. Price, Freya Shearer, Mark Stoove, Kathryn Young, Yanqin Zhang, Katherine B. Gibney, Margaret Hellard

**Affiliations:** 1 Melbourne School of Population and Global Health, The University of Melbourne, Carlton, VIC 3053, Australia; 2 The Burnet Institute, Melbourne, VIC 3004, Australia; 3 Coelho Networks, Melbourne, VIC 3058, Australia; 4 Peter Doherty Institute for Infection & Immunity, The University of Melbourne, Parkville, VIC 3052, Australia; 5 School of Public Health and Preventive Medicine, Monash University, Melbourne, VIC 3004, Australia; 6 Australian Research Centre in Sex, Health and Society, La Trobe University, Bundoora, VIC 3083, Australia

**Keywords:** pandemic, culturally and linguistically diverse, migrant, research processes

## Abstract

Participation of people from culturally and linguistically diverse (CALD) communities in public health research is often limited by challenges with recruitment, retention and second-language data collection. Consequently, people from CALD communities are at risk of their needs being marginalised in public health interventions. This paper presents intrinsic case analyses of two studies which were adapted to increase the cultural competence of research processes. Both cases were part of the Optimise study, a major mixed methods research study in Australia which provided evidence to inform the Victorian state government’s decision-making about COVID-19 public health measures. Case study 1 involved the core Optimise longitudinal cohort study and Case study 2 was the CARE Victorian representative survey, an Optimise sub-study. Both case studies engaged cultural advisors and bilingual staff to adjust the survey measures and research processes to suit target CALD communities. Reflexive processes provided insights into the strengths and weaknesses of the inclusive strategies. Selected survey results are provided, demonstrating variation across CALD communities and in comparison to participants who reported speaking English at home. While in most cases a gradient of disadvantage was evident for CALD communities, some patterns were unexpected. The case studies demonstrate the challenge and value of investing in culturally competent research processes to ensure research guiding policy captures a spectrum of experiences and perspectives.

## 1. Introduction

Rapid research to monitor COVID-19 infection status and behaviours has been a critical tool for government to inform public health policy and control the virus [1]. Research investigating different understandings, values, attitudes and experiences of social, mental health and economic impacts is also required to guide decision making [2]. However, such research is limited if it is not inclusive of diverse communities and experiences. In Australia, individuals from culturally and linguistically diverse (CALD) communities are less likely to be included in health research for various overlapping reasons, including language barriers, mistrust of research, stigma, lack of access to information, exclusion and othering [3,4,5,6,7,8]. CALD remains a broad and often reductive term used in Western academia that aims to describe migrants who have come from generally non-English speaking countries to a Western, Educated, Industrialised, Rich and Democratic (WEIRD) country in which the research is taking place [9]. In many countries across the world, the COVID-19 pandemic worsened existing economic and health-based inequalities. The people who felt these worsening inequalities were those who were already experiencing disadvantage [10,11]. Including CALD communities in COVID-19 research is critical because they have been shown to be at increased risk of COVID-19 infection and the serious morbidity and mortality that can arise from it [5,12,13,14]. They are also likely to be more vulnerable to adverse impacts of pandemic related restrictions [15,16,17,18]. These kinds of health inequalities and barriers to research can be understood in combination through the lens of intersectionality, where inequalities are the result of a range of overlapping processes and structures related to racism and discrimination [19].

Even before the pandemic, CALD groups were more likely to have experienced discrimination, potentially resulting in low levels of trust in authorities [20]. During the pandemic they were more likely to live in conditions with limited capacity to physically distance and quarantine or isolate as required [21,22] and be employed in precarious but essential occupations highly exposed to the virus such as cleaning, food production and delivery, aged care and childcare [23,24]. In Australia, those on temporary visas such as people seeking asylum and international students were explicitly excluded from a range of government supports during the pandemic, leaving them at risk of destitution if suddenly unemployed and therefore less able to stay home if exposed [25,26,27]. Some were also ineligible for government funded healthcare [28,29,30]. Furthermore, information about COVID-19 was not always readily available in community languages and, even when translations were provided, may not have been accessible due to exclusive use of mainstream media for dissemination and lower levels of literacy and health literacy among the target communities [16,31,32].

In this paper we present two case studies from the Optimise study conducted in Victoria, Australia, in which research processes were substantially adapted to ensure greater inclusion of the experiences of Victorians from CALD communities, including those initially precluded from participating because of language barriers. Study adaptations were consistent with a cultural competence in research approach [33] in which “high quality research takes into account the culture and diversity of a population when developing research ideas, conducting research and exploring the applicability of research findings” (p. 6). The process included close involvement of members of culturally diverse communities as staff members, advisors and collaborators in the development and conduct of the research. The intention was to provide policy decision makers with a more accurate description of the experiences of the Victorian population and improve the utility of the data for providing indications of how diverse communities were understanding and experiencing COVID-19 response measures.

### 1.1. Victorian Context

Among Australian states and territories, the state of Victoria was the hardest hit by COVID-19 outbreaks early in the pandemic and, as a result, at the time of writing in late 2022 it remained the state with the highest death toll of any in Australia, closely followed by New South Wales [34]. Prior to vaccine availability, Victoria experienced a number of significant COVID-19 outbreaks. As a result, Victoria also had the most stringent and extended public health responses aimed at controlling COVID-19, comprising a mix of restrictions that were adjusted over time including the closure of non-essential businesses, stay-at-home directions, restriction of movements to within a 5 km radius of home, border closures, isolation following actual or potential exposure, mask wearing, physical distancing, contact tracing measures and vaccination requirements for access to certain businesses and services [35,36,37]. Application of these mitigation strategies varied over time and location (e.g., state-wide; metropolitan versus regional; selectively applied to high risk local government areas and premises). In a particular high-profile example, nine public housing residential towers were placed into immediate hard lockdown in response to 24 residents of the towers testing positive to COVID-19 in July, 2020 [38,39]. A large proportion of people residing in publicly funded housing in Victoria are from CALD backgrounds. The implementation of this hard lockdown without initial community engagement or planning for the diverse needs of the residents resulted in significant hardship for the residents and caused broader community concern [40,41]. This example highlights that inequity occurs when efforts to reduce the spread of COVID-19 are implemented without consideration for the social, economic and health needs across a highly diverse society [42,43].

### 1.2. The Optimise Study

The Optimise study was designed to provide evidence to improve government decision making regarding the Victorian COVID-19 response, both to prevent new infections and to reduce the health, social and economic impacts of COVID-19 public health measures (https://optimisecovid.com.au/, (accessed on 20 November 2022)). It was a mixed method study undertaken in partnership between Burnet Institute and Doherty Institute, in collaboration with University of Melbourne, Swinburne University of Technology, Monash University, La Trobe University, Murdoch Children’s Research Institute, the Centre for Ethnicity and Health, Social Network Analysis (SNA) Toolbox, Coelho Networks and the Health Issues Centre.

There were several components to the Optimise study. The central component was a longitudinal cohort study of Victorian adults and their social networks. Certain population groups were oversampled because they were considered to be at risk of: (i) contracting COVID-19; (ii) developing severe COVID-19; (iii) experiencing negative impacts of restrictions introduced to reduce COVID-19 transmission and/or (iv) having greater difficulty understanding or following Government restrictions. Participants completed a schedule of repeated questionnaires and online diaries and a small subset of the Optimise cohort participated in qualitative interviews at key time points. There were additional related studies under the umbrella of the Optimise research program, including the CARE (COVID-19 Attitudes, Resilience and Epidemiology) survey which was led by University of Melbourne and Doherty Institute.

The first case study presented below involved the Optimise cohort and the second case study involved the CARE survey.

## 2. Case Analysis Methods

An intrinsic case analysis approach [44] has been applied to the following two case studies to explore in depth the benefits and struggles of adjusting an existing study design to include data collection in languages other than English among people from migrant backgrounds during a global pandemic.

### 2.1. Case Selection

These cases have been selected as they were both pre-existing studies based in Victoria, Australia, that were re-designed to expand data collection methods during the initial waves of COVID-19 infection to specifically engage culturally and linguistically diverse participants. These adaptations represent intrinsic cases, where the novelty of adaptations may provide real value to future research and policy.

### 2.2. Case Analysis

Each case analysis explores;

1. The original study design followed by adaptations made to engage CALD participants.
2. Sample characteristics taken from quantitative surveys.
3. Quantitative survey results of participants who were recruited using original study mechanisms and those who were recruited using adapted study mechanisms (e.g., bi-lingual data collectors).

Each case is assessed for successful engagement in the study through recruitment, survey completion and retention. Additionally, comparisons between CALD and non-CALD groups are made. Case study 1 includes additional qualitative reflections from bi-lingual data collectors. These reflections were gathered from short [10–15 min] unstructured interviews with the Optimise bi-lingual data collectors, where data collectors were asked to reflect on how effective protocol adaptations were in overcoming barriers to research. Both cases draw on data collector insights to describe the contexts behind the quantitative findings.

## 3. Case Analysis Results

### 3.1. Case Study 1—Expanding the Optimise Longitudinal Cohort

#### 3.1.1. Study Design & Adaptations

Data collection for the Optimise cohort began in September 2020 and concluded at the end of August 2022. The study focused on how people in the community were experiencing government measures to control COVID-19 (e.g., early testing, self-isolation and quarantine, stay-at-home measures) and identifying factors that influenced adherence including unintended health, social and economic consequences. Data collection involved a combination of validated scales and measures developed in response to pandemic-related issues as they emerged. Participants were also asked to list people who play an important role in their life (herein referred to as ‘key people’) as part of a social network recruitment and analysis approach. ‘Key people’ were defined as those who were part of the participants’ life on a daily or weekly basis, such as family, friends, partners, housemates, neighbours and/or co-workers. Survey responses were analysed to assess how experiences differed between individuals and groups over time. The results were submitted in a series of rapid reports to the government to inform policy and service delivery decision making. Results are all made publicly available in English, Arabic and Simplified Chinese [https://optimisecovid.com.au/study-findings/] (accessed on 20 November 2022).

The Optimise study initially collected data in English only but still recruited 61 participants with CALD backgrounds through this process. Funding was secured in late 2021 to expand the study for increased participation from people from culturally diverse backgrounds who were less likely to be able to participate in English and to be reached through the general recruitment processes. Bilingual data collectors were employed and began by helping to revise the participant recruitment and data collection protocol to increase accessibility for research participants from CALD communities and specifically attempt to include those not fluent in English. The five bilingual data collectors included two Arabic speakers, two Mandarin speakers and one Dinka speaker. The resources did not allow coverage of all migrant language groups, so these were chosen as representative of large numbers of non-English speakers in Melbourne and as groups experiencing particular disadvantage during the pandemic, for example, due to racism, temporary visa status, exclusion from pandemic economic support measures and/or social disadvantage.

The bilingual data collectors were initially trained in the existing study protocol and English-based data collection methods. Workshops were then held to discuss the viability and socio-cultural appropriateness of existing methods for capturing data in different languages and how they would overcome the unique barriers to research participation for different CALD groups. An external facilitator with expertise in organisational cultural competence was brought in to guide the review of engagement and retention strategies. The revised protocols and study materials were further tested and refined during the data collection period. A debriefing session at the end of the recruitment and data collection period was also conducted to allow the Optimise team to identify and record barriers experienced during the study.

This method of recruitment and data collection, in line with cultural competence in research approach, aimed to promote knowledge exchange within and between communities and university/research institutes involved in driving policy [33,45]. It enabled a more nuanced approach to data collection, taking into account community health literacy levels, health belief systems and a collectivist viewpoint of health in general.

#### 3.1.2. Sample Characteristics

A total of 94 participants from CALD communities approached by bi-lingual data collectors using the above targeted/inclusive methods consented to participate in the study from September 2021–December 2021. Of those, 88 participants completed baseline surveys. The demographic details of the newly recruited CALD participants are provided in Table 1. We note that these new CALD participants added to the existing 691 participants in the Optimise cohort study which included 61 (8.8%) people from CALD communities who spoke a language other than English at home but were recruited using the general Optimise recruitment processes and participated in English.

Arabic speakers made up almost half of the new CALD recruitments, Sudan and China were the most common countries of birth, almost half were aged between 35 and 54 and the majority were female, COVID-19 negative at baseline, university educated, permanent residents, not actively religious and living in metropolitan areas.

The demographics presented in Table 1 demonstrate that the adapted strategies were effective in recruiting a group of people across a range of ages and languages spoken at home. In addition to this, employing snowball sampling using bilingual data collectors was highly successful in rapidly recruiting participants over the course of three months (September–December 2021). In comparison, the recruitment of the 55 CALD participants who joined the study prior to protocol revisions took 13 months (October 2020–November 2021), demonstrating how difficult it is to reach CALD communities through standard research advertising and recruitment methods. However, the permanent residency status and the high education level of the majority of the participants suggest that more marginalised groups were still not well represented.

#### 3.1.3. Quantitative Survey Results

Key survey findings relating to COVID-19 diagnosis, experiences of racism and mental health are provided below. Social and participation indicators are presented in Table 2, showing the average number of key people nominated by the newly recruited CALD participants and the proportion who withdrew from the study at some point after completion of at least baseline measures, compared to the existing Optimise participants recruited using mainstream methods. There was a lower number of average key people nominated by participants in all CALD groups compared with the existing Optimise participants who reported speaking English at home. There was also a higher proportion of withdrawals among Arabic and Dinka speaking participants.

**Figure 1:**
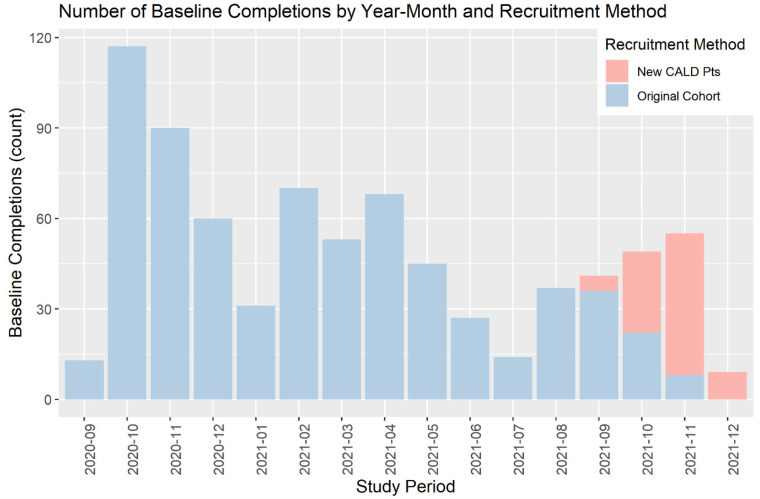
Describes the number of participants who completed baselines in each month. Colours distinguish participants who were recruited as part of the original cohort and those who were recruited after protocol revisions to target recruitment of CALD participants.

Baseline survey results are presented for CALD participants who were enrolled after protocol revisions. Due to rolling recruitment, completion of baseline surveys was spread over an extended period where baseline measures for original Optimise participants were completed from 25 September 2020 through to 27 November 2021, while the baseline measures for targeted CALD communities were completed from 9 September 2021 through to 7 December 2021. This rolling recruitment is illustrated in Figure 1. In order to present comparable data for COVID experiences, data from the newly recruited CALD participants captured in their baseline surveys were compared with data from the original Optimise participants captured in follow-up surveys completed over a similar time period, i.e., between 9 September 2021 and 4 December 2021 (see Table 3).

Table 4 presents survey and diary completion rates and so includes full results from original Optimise participants collected from 25 October 2020 through to the end of the study in August 2022 and from newly recruited CALD communities collected from 9 October 2021 through to August 2022.

The trends in the results show variability across CALD groups on different measures, specifically a higher proportion of people from Arabic speaking communities reported COVID-19 infections, a higher proportion of those from Dinka speaking communities reported knowing someone who had been infected and a higher proportion of people from Chinese communities reported experiencing racism. A higher proportion of people from English speaking background reported symptoms of anxiety. Further, the results in Table 4 show a low follow-up completion rate in Arabic and Dinka participants, but very high completion rate among Chinese participants. Among Dinka participants who withdrew from the study, most did so before completing any surveys. The intention here is not to provide a comprehensive account of the study results but rather to present some selected results from the original Optimise cohort and participants from CALD groups who were recruited using adapted inclusive strategies.

#### 3.1.4. Qualitative Staff Reflections

Data collection for this cohort commenced during one of the extended periods of lockdown which impacted upon the recruitment process. Ideally, in-person recruitment of these three language groups would have been preferred. However, the approaches were tailored to the extenuating situation and the bilingual data collectors made every effort to make participants feel as comfortable as possible when participating in the absence of face-to-face data collection through regular contact, flexibility of survey completion methods (self-completion/interview support) and sharing of study results in the appropriate language.

The staff debriefing sessions identified a range of barriers and facilitators to recruitment and participation, either experienced by the data collectors themselves or reported to them by the participants. Barriers and facilitators are presented below as common themes, with specific details provided where relevant for each of the Arabic, Mandarin and Dinka speaking communities.

##### Reaching Potential Participants

There was mixed success with the strategies used in Case Study 1 to reach potential participants. The pre-existing networks of the data collectors, both personal and professional, were helpful, particularly when pandemic public health measures required mask wearing or prevented any face-to-face engagement. Data collectors also made specific attempts to extend existing networks to include a wider range of participants. Particularly among the Arabic community, networks were extended to attempt to include participants from outside of the Sudanese community. Attempting to use other means to reach community organisations such as phone calls and emails were not successful for the Chinese community, perhaps because these organisations were also affected by public health measures and there was temporary cessation of group activities. However, the use of online platform WeChat was an effective method for recruiting and disseminating study findings among participants from Chinese backgrounds.

For all CALD groups, many of their nominated key people were based overseas and therefore were not eligible for recruitment, limiting the potential for snowball sampling.

##### Motivation to Participate

It emerged that the translation of materials and the use of bilingual data collectors was important to ensure participants understood what was involved and were able to easily express interest. It also reinforced the relevance of the research for their community and the fact that their views and experiences were valued.

Many participants from the Chinese community were international students engaged with local universities and therefore had an understanding of how Western research paradigms worked. COVID-19 was also regularly reported on in Chinese media which may have affected the relatively high engagement and retention of Mandarin speakers in the Optimise study.

##### Privacy Concerns

Even when participants from CALD communities were keen to be involved in the study, they told data collectors that they were hesitant to share details about key people in their lives because of privacy concerns. People also did not feel comfortable having the data collector contact their key people until they had a chance to speak with them. This was generally consistent with the experiences of data collectors recruiting key people networks for the original Optimise participants prior to the protocol revisions. Some participants became more comfortable sharing details about the key people in their lives when the data collector explained how the data would be used and why it was important to collect such information.

Among the Arabic and Dinka speaking groups, many were unfamiliar with longitudinal study designs. The repeated personal questions that were asked because of repeat waves of the longitudinal survey raised suspicions that the data was being used “for something else”. For this reason, one participant indicated that they had not provided accurate data about themselves.

Many participants in the Dinka speaking and the Chinese community (especially the elderly) also spoke to data collectors of privacy concerns when sharing their residential address and bank account for reimbursements each time they contributed data. Dinka speaking participants did not put value on reimbursement of their time for participation, many did it for the greater good and to inform policymakers and pandemic management policies.

##### Continued Participation

Data collectors noted that research fatigue was an issue for many CALD participants due to the repeat measures that were part of the Optimise longitudinal cohort design. The occasional snapshot surveys that asked questions about current COVID-19 related issues were preferred instead of the repeat waves of the longitudinal survey. Other barriers to continued participation included return to work and change in daily routine during the Holy Month of Ramadan.

Factors which seemed to support ongoing participation included commitment to informing policy decision making, having more time available during stay-at-home periods, and personal connection with the data collector. This connection provided a sense of social support, trust and personal commitment as indicated by this comment to a data collector from a participant in the Arabic speaking group—“I do this for you, brother, just for you”.

### 3.2. Case Study 2—Expanding the CARE Cohort

#### 3.2.1. Study Design & Adaptations

The CARE survey was a series of cross-sectional online surveys of attitudes, behaviours and experiences relating to COVID-19 risks, public health measures and the mental health and wellbeing impacts of the pandemic. Data collection involved a combination of validated scales and measures developed in response to pandemic-related issues as they emerged. It was conducted periodically to capture the impacts of changes in disease transmission and public health measures. The surveys conducted for Optimise involved a Victorian representative sample and were conducted in September 2020, July 2021, September 2021, December 2021 and April 2022. Some of the survey items remained consistent for each wave of the survey, others were adjusted to capture emerging issues. The questionnaire was administered online by YouGov Australia (https://au.yougov.com/) (accessed on 20 November 2022) on behalf of University of Melbourne. YouGov regularly administers surveys of public opinion to a panel of over 120,000 Australian adults. Proportional sampling quotas ensured that the final sample represented the Victorian adult population based on age, gender, metropolitan/regional residence, multicultural background and household income. The responses were then weighted according to census data.

In 2020 additional funding was secured to include a complementary research process for the September 2020 wave of the CARE survey. A multicultural organisation, the Centre for Culture Ethnicity and Health (CEH), was engaged as a research partner to provide cultural competence guidance and research assistance. Bilingual staff within CEH were trained in the existing study protocol and English-based data collection methods, and helped to reshape the research processes and survey questions to ensure suitability for CALD participants. They then collected the data and contributed to data analysis and reporting. The lead consultant (AC) made the greatest contribution throughout and also acted as an advisor to Case Study 1.

The benefit of engaging CEH for help with recruitment and data collection for the bilingual interviewer-assisted surveys was that they were already embedded in the target communities as a leading organisation in multicultural health. They utilised their existing community networks to engage with potential participants, supplemented by social media and a combination of snowball and purposive sampling to seek diversity within the culturally diverse sample.

The consent process had to be handled carefully and was time intensive, involving advance phone calls, sending information about the research for potential participants to consider and reassuring community leaders and other family members to build trust and understanding. It was a much longer process than is typically involved when recruiting participants from English-speaking backgrounds. The interviewer read the research information and consent forms to participants in their language of choice rather than relying on translated materials. This was done to address literacy issues, particularly for some Dinka speakers given that Dinka is primarily an oral language. The standard list of support services that was provided in advance to all CARE participants, was expanded to include more services that were targeted to culturally diverse communities such as the InTouch Multicultural Centre against Family Violence and Study Melbourne which provides a range of support services for international students.

CEH staff suggested adjustments to the survey questions to ensure relevance for the CALD participants. This resulted in the addition of questions relating to visa and residential status, dwelling type, experiences of racism, understanding of public health advice, income security and need for/access to financial assistance.

It was important that the bilingual interviewers understood the nature and value of the research because they needed to be able to explain this to the participants. When they realised the Optimise and CARE research was being used to guide government decision making, they were enthusiastic about the research and the opportunity it provided for CALD communities to have influence and protect their communities.

In-person interviews would have been preferred but were generally not possible because of the public health measures in place at the time. Interviewing by phone was difficult for the interviewers because they had to concurrently enter the data into the online survey on behalf of the participant, so Zoom was typically used as the interview platform. This had the added benefit that the share screen function enabled the participant to see their responses being typed directly into the survey in English, building trust in the interviewer.

CEH advised that it was important to provide reimbursement for participants’ time because many were in precarious financial circumstances. Cash was preferred because many people are unsure of how vouchers and electronic gift cards work. However, because of organisational accountability systems, electronic gift vouchers to major stores were used that could be sent by email or text.

The bilingual interviewers identified some issues with the way certain survey questions were worded that were understood differently by participants depending on their sociocultural framework. For example, there was a very different understanding of the concept of mental health for many participants, particularly older participants, and so some of the questions were difficult for them to understand and to respond to. Mental health was often understood as related to being ‘crazy’, but not mood related. Participants expressed frustration at the perceived repetition of questions, particularly those related to mental health, perhaps not understanding how the questions differed, but also likely reflecting the response burden of the survey as a whole.

Insights were gained into issues that were not captured by the survey but which became apparent through the interviewing process. These issues included: the challenges of isolating in crowded housing; fear of COVID-19 testing because of the stigma associated with positive results in the early stages of the pandemic; and public measures that reinforced discrimination such as the instant hard lockdown for some participants in high rise public housing, triggering mistrust in authorities compounded by past refugee experiences.

It was sometimes problematic interviewing more than one person from the same household or even conducting the interview when others were at home. Sometimes young people would not answer if their parents were in the room or women would wait until their male partners left to go to the shops before they felt comfortable being interviewed. Indications of controlling behaviour and potential family violence emerged in some instances. This highlighted the benefit of providing information about family violence services to all participants to reduce the possibility that it would raise the suspicion of partners, which may have increased risk to participants. It also highlighted the benefit of partnering with an agency (CEH) with relevant expertise and with support services for participants and the interviewers.

#### 3.2.2. Sample Characteristics

The September 2020 wave of the online CARE survey was completed in English by 1006 Victorians from the 7 to the 15 of September, including 700 people who reported that they speak only English at home and 303 people who reported speaking a language other than English at home (as a proxy for people from CALD backgrounds). An additional three people declined to respond to the question about language.

The additional concurrent bilingual interviewer-assisted surveys were completed in September/October 2020 by 146 adults from CALD groups that were identified as being at higher risk of infection, discrimination and/or disadvantage from pandemic restrictions in the early stages of the pandemic: Dinka speakers (48), Arabic speakers (48) and international students (50). These interviews were conducted over Zoom by a bicultural worker speaking in the respondents’ language of choice.

#### 3.2.3. Quantitative Survey Results

**Figure 2:**
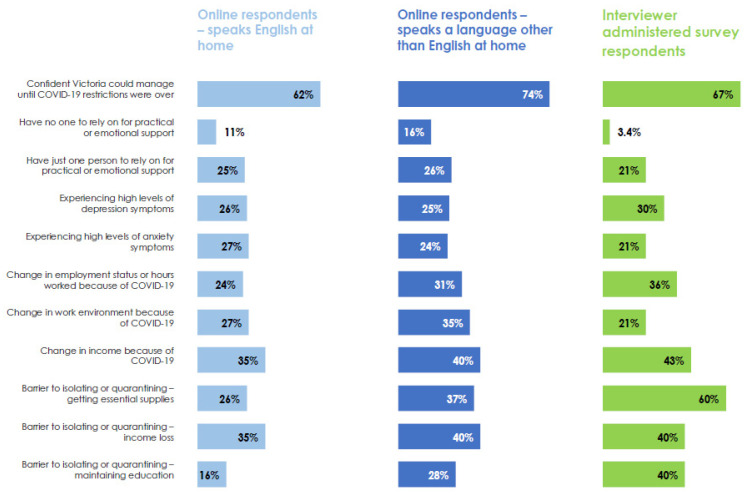
Comparison of CARE responses for different participant groups, Victoria, September–October 2020.

The CALD survey findings showed both similarities and differences in responses for (1) online survey respondents who speak English at home (completed from 7–15 September 2020), compared to (2) online respondents who speak another language at home (but completed the surveys online in English from 7 to 15 September 2020) and (3) bilingual interviewer-administered survey respondents (completed from 22 September to 14 October 2020), as shown below in Figure 2. Some of the responses show a gradient of disadvantage with the interviewer-administered survey respondents at greatest risk, particularly in relation to barriers to complying with quarantine and isolation measures. This highlights the importance of using additional strategies to reach groups who may be more marginalised in traditional research participation and are at greater risk from collective emergencies such as a pandemic.

## 4. Discussion

Rapid research has made a critical contribution to responsive decision making about COVID-19 public health measures [1]. However, too often general population studies do not include high risk CALD groups and may miss important intersecting factors that can lead to health inequality [19]. The results from the Optimise study demonstrate that making adaptations to standard study designs to overcome structural barriers to research allow exploration into the intersection between health, race, language and culture. This is exemplified in the finding that Mandarin participants report higher experiences of COVID-19 racism, or that all CALD participants reported better mental health scores than non-CALD participants. The case studies above demonstrate that standard research practices do not do enough to capture participants of diverse experiences.

These inclusive approaches do require additional resources and often more time in establishing partnerships and research processes. However, benefits are likely to include more accurate findings related to health inequality, improving the quality of information available to policy makers and appropriate allocation of support services, ultimately improving health outcomes [19].

The initial reflexive processes described for each case study identified a range of existing research processes that unintentionally excluded CALD participants and survey items that did not account for their circumstances. These differences in lived experiences compared to non-CALD participants were reinforced by the research findings which showed the CALD communities were reporting greater impacts from some aspects of the COVID-19 experience compared to online survey participants. This aligns with the limited existing research about higher risks for CALD communities [12,23,42] and illustrates the importance of inclusive research methods and the limitations to quantitative data if recruitment and data collection methods do not include marginalised groups. The survey findings also showed ways in which the different CALD groups were showing variable impacts from COVID-19 at certain timepoints such as experiences of racism.

Using the case analysis approach, particularly through collecting additional staff reflections, exploration beyond quantitative responses into how health research intersects with broader parts of life became possible. Findings such as how impactful written translations of the research documents were for participants in Case Study 1 was illuminated through this process. Translations are perhaps an obvious inclusion in COVID-19 communications and research materials but are not necessarily straightforward to implement [16,31,32]. While the written form of the survey allowed accuracy checking beforehand and consistency in use, some problems with this approach were identified when the bilingual interviewers read and explained the materials. Some CALD communities (e.g., some Dinka speakers) rarely used their language in written form and therefore required a verbal explanation. Other languages had a wide range of dialects and/or required different phrasing depending on the social and religious customs, gender and topic sensitivities for the research participants. The bilingual interviewers needed to adjust for these variations to ensure cultural competence, shared understanding and consistency in interpretation of the study questions.

The adaptations for each case study reflect a commitment to progressing towards cultural competence in research processes [33]. There were still challenges in recruitment and sampling, particularly because the pandemic restrictions prevented many of the planned in-person methods. The subsequent reliance on on-line recruitment methods meant that a younger cohort was reached, with many of the participants being able to speak English.

Trust was a critical factor in recruitment. In a diasporic context, where there is a general unfamiliarity with taking part in longitudinal research and more broadly western paradigms of research, it was not easy to convince some participants that taking part in such research would be of benefit to their own communities and to the wider society. Suspicious thoughts following exposure to myths about the origins of COVID-19, public health measures, research, testing and vaccines were common. This was identified in an international literature review as a common problem that can arise from reliance on social media information sources and is a potential barrier to engagement in preventative health measures [47].

Purposive snowball sampling was helpful as a recruitment method for both studies as it relied on reaching participants through trusted organisations and their own social networks. In Case Study 1, these recruitment methods aided rapid recruitment in Arabic, Chinese and English-speaking communities. However, we observed that it was ineffective in Dinka-speaking communities. This may be due to the influence prospective participants have on one-another within a social network. For example, in a close-knit community, if one participant drops out or aligns themselves against the research, their contacts may be influenced to drop out or reject attempts at recruitment. This effect may have been accentuated for Dinka communities [48].

Other barriers to recruitment, particularly of key people, included privacy concerns, limited local social networks, recruitment occurring when COVID restrictions were lifting and the underlying perception that western research organisations do not have their best interests in mind. Barriers to CALD participant retention included limited understanding of longitudinal research and the need for repeat waves of data; data collection occurring when lifting of restrictions was allowing return to work and therefore less time for research participation; and limited understandings of western healthcare. This suggests that, despite dedicated efforts to identify and overcome barriers in the recruitment and retention of study participants from diverse backgrounds, some structural limitations persisted. The insights shared through these case analyses were captured through internal staff reflexive processes rather than collected and analysed as research data. Inclusion of formal analyses of cultural competence in future population health research studies is needed to address the gap in evidence [33]. A more developed cultural competence approach, using a collective contribution to interpretation of findings and co-development of immediate direct outcomes, may also have been more engaging and empowering for participants and have led to additional valuable insights.

Finally, these case analyses make a clear argument towards ensuring that data-driven and community lead public health policy must make a considered effort to consult, partner and engage with CALD communities from the beginning of research. It is argued that, without such community engagement or participatory research, processes important experiences of health inequality are not captured. Danchin and colleagues suggest that aligning public health policy with public sentiment leads to better adherence, therefore if public sentiment is appropriately measured, policy should be more likely to succeed [49].

## 5. Combined Limitations

For both case studies, people from particular CALD groups were recruited to provide indicative insights into their COVID-19 experiences, recognising that they may be facing additional challenges compared to participants who speak English at home or those well acclimatised to Australian cultural norms. The recruitment of these subgroups was not representative of each distinct CALD community or language group and hence results should not be interpreted in that way. It is also possible that participants belonged to more than one of the nominated risk groups (e.g., an international student from the Chinese community), consistent with the issue of intersectionality and its influence on heightened risk of health inequality.

In the CARE case study, it is important to consider demographic differences when comparing different subgroups. Both the CALD interviewees and the online sub-group who speak a language other than English at home were considerably younger on average than the online English-only sub-group. Younger participants may be more likely to be international students and/or casual workers and therefore more vulnerable to life disruptions and loss of income or work hours. It is also acknowledged that language spoken at home is an imperfect proxy to identify a CALD subgroup (one of the indicators used for Optimise and CARE online participants).

The intrinsic case analysis approach utilized in this paper is relatively simple in order to draw readers’ attention to the methodological adaptations applied in each case. Any comparative results reported should be considered indicative only. Further research including more comprehensive case analysis techniques is needed to test the study findings.

## 6. Conclusions

Culturally competent research using deliberate strategies to reach CALD communities typically marginalised from research provides important data to increase the inclusivity of public health measures and to address pre-existing inequities. Additional targeted strategies to include CALD communities require additional time, resources, planning and partnership with the communities being researched. Both cases presented in this paper demonstrate how deliberate efforts to increase the cultural accessibility, appropriateness and safety of the research enabled CALD communities to inform Victorian Government policies and were made publicly available for wider use (https://optimisecovid.com.au/news/) (accessed on 20 November 2022). The case studies demonstrate that major revisions to study designs are necessary to effectively collect accurate data among CALD communities. The adapted research processes identified both research challenges and additional pandemic risks for CALD participants compared to non-CALD participants. Some results also showed unexpected patterns of difference in COVID-19 impacts. This demonstrates the limitations and exclusionary nature of standard population research studies conducted by universities, namely recruitment mechanisms that target educated English-speakers, reliance on digital reimbursement and the assumption that western research paradigms are universally understood, but in fact are only appropriate for participants from WEIRD societies. Further, this case analysis demonstrates the need to consider the concept of intersectionality of health and social inequalities. Both studies were limited in the extent to which the CALD participants represented their broader community and the notion that CALD as a “catch-all” term is reductive to the diverse experience of recent migrants in Australia. Without specific consideration, it is easy for public health strategies to be guided only by the experiences of those at least risk in the community, particularly if the groups most at risk are systematically excluded from the research. It is recommended that public health research funding bodies provide adequate support to ensure these groups are fully represented in research, as opposed to being included only if additional funding becomes available. Policy derived from this type of research should always ensure people experiencing health inequality are represented and overlapping factors that contribute to this health inequality are adequately described.

## Figures and Tables

**Table 1:** Participant Demographics recruited by bi-lingual data collectors reported as count (%).

		Newly Recruited CALD Participants	Existing Optimise Cohort
Age Category (Years)			
	Missing	6	2
	18–24	16 (18.2%)	121 (17.6%)
	25–34	15 (17.0%)	140 (20.3%)
	35–44	23 (26.1%)	98 (14.2%)
	45–54	23 (26.1%)	110 (16.0%)
	55–64	9 (10.2%)	118 (17.1%)
	65–74	2 (2.3%)	80 (11.6%)
	75+	0 (0.0%)	22 (3.1%)
Language Spoken at Home			
	Arabic	48 (51.1%)	0 (0.0%)
	Mandarin	33 (35.1%)	5 (0.7%)
	Dinka	13 (13.8%)	0 (0.0%)
	Cantonese	0 (0.0%)	4 (0.6%)
	Filipino	0 (0.0%)	5 (0.7%)
	Vietnamese	0 (0.0%)	4 (0.6%)
	Hindi Urdu	0 (0.0%)	15 (2.2%)
	Other ^†^	0 (0.0%)	28 (4.1%)
	English	0 (0.0%)	630 (91.2%)
Sex			
	Female	60 (63.8%)	499 (72.2%)
	Male	33 (35.1%)	187 (27.1%)
	NA	0 (0.0%)	5 (0.7%)
Country of Birth			
	Missing	6	0
	Australia	8 (9.1%)	495 (71.6%)
	Saudi Arabia	2 (2.3%)	0 (0.0%)
	South Sudan	8 (9.1%)	0 (0.0%)
	Sudan	24 (27.3%)	0 (0.0%)
	China	23 (26.1%)	10 (1.4%)
	Eritrea	12 (13.6%)	0 (0.0%)
	Other African Country ^†^	2 (2.3%)	13 (1.9%)
	Other Middle Eastern Country ^†^	4 (4.5%)	2 (>0.01%)
	Chinese speaking countries outside of China ^†^	5 (5.7%)	2 (>0.01%)
	Other English speaking country	0 (0.0%)	79 (11.4%)
	Other country where English is not the primary language	0 (0.0%)	90 (13.0%)
	Prefer not to say	0 (0.0%)	0 (0.0%)
Highest Level of Education completed			
	Missing	6	0 (0.0%)
	Primary school	8 (9.1%)	31 (4.5%)
	Highschool	9 (10.2%)	95 (13.7%)
	TAFE/trade	16 (18.2%)	110 (15.9%)
	Undergraduate	33 (37.5%)	257 (37.2%)
	Postgraduate	20 (22.7%)	193 (27.9%)
	Prefer not to say	2 (2.3%)	5 (0.7%)
Current Residential Status			
	Missing	6	0
	Permanent resident/Australian citizen	68 (77.3%)	636 (92.0%)
	Other	20 (22.7%)	55 (8.0%)
Active Member of Religious Group or church			
	Missing	6	0
	No	64 (72.7%)	611 (88.4%)
	Yes	19 (21.6%)	73 (10.6%)
	Prefer not to say	0 (0.0%)	7 (1.0%)
Residential Location			
	Missing	7	6
	Metropolitan Melbourne	81 (93.1%)	547 (79.9%)
	Regional Victoria	6 (6.9%)	138 (20.1%)

^†^ To protect participants’ anonymity, small group sizes have been combined into aggregate groups.

**Table 2:** Survey participation of new versus existing CALD participants.

Average Number of Key People Nominated: Mean * (SD)	
Arabic speakers	3 (2.0)
Mandarin speakers	5 (3.0)
Dinka speakers	4 (2.3)
Existing Optimise participants (speak LOTE ^†^ at home)	8.47 (4.7)
Existing Optimise participants (speak English at home)	10.7 (5.7)
Number Withdrawn: *n*/N (%)	
Arabic speakers	11/48 (22.9%)
Mandarin speakers	4/33 (12.1%)
Dinka speakers	5/13 (38.5%)
Existing Optimise participants (speak LOTE ^†^ at home)	11/61 (18.0%)
Existing Optimise participants (speak English at home)	106/630 (16.8%)

Note: Several existing participants withdrew after consent and did not complete a baseline or follow-up survey. These participants contribute to the total number of participants withdrawn, but do not contribute to the 691 participants specified in section: Section 3.1.2 Sample Characteristics. * Rounded to the nearest whole number (person). ^†^ Language other than English.

**Table 3:** COVID experiences by language group.

	Language Spoken at Home										
		Newly Recruited CALD Participants						Existing Optimise Study Participants			
		Arabic (*n* = 43)		Dinka (*n* = 12)		Chinese (*n* = 33)		LOTE (*n* = 50)		English (*n* = 549)	
		Number of pts.		Number of pts.		Number of pts.		Number of pts.		Number of pts.	
Ever Tested positive to COVID ^1^	Yes	4	9.3%	1	8.3%	0	0%	17	34.0%	154	27.8%
	No/Prefer not to say	39	90.7%	11	91.7%	33	100%	33	66.0%	399	72.2%
Know someone who has been diagnosed with COVID-19	Yes	24	55.8%	12	100.0%	6	18.2%	38	76.0%	527	95.3%
	No	17	39.5%	0	0%	26	78.8%	12	24.0%	26	4.7%
	Prefer not to say	2	4.7%	0	0%	1	3%	0	0%	0	0%
Experienced or Witnessed COVID-19 related racism	Yes	8	18.6%	2	16.7%	16	48.5%	22	44.0%	205	37.1%
	No	35	81.4%	10	83.3%	17	51.5%	28	56.0%	348	62.9%
GAD-7 Scores (mean, SD)	Mean	5.4	-	3.4	-	3.4	-	4.82		5.92	-
	SD	4.9	-	4.3	-	4.0	-	4.92		5.27	-

^1^ A brief 7 item self-report measure of General Anxiety Disorder [46]; Footnote: Data for the first three variables are presented as counts and percentages, where the denominator is the total number of participants in each language group. LOTE—language other than English.

**Table 4:** Survey and diary completion rates of participants in each language group among those remaining active in the cohort until the end of the study and from those withdrawing before the end of the study.

		Arabic		Dinka		Chinese		Existing (spk LOTE at Home)		Existing (spk English at Home)	
		M	IQR	M	IQR	M	IQR	M	IQR	M	IQR
		*n* = 36		*n* = 8		*n* = 29		*n* = 50		*n* = 524	
**Active**	Follow-up Survey	35.4%	12.2, 72.3	14.3%	9.38, 19.6	87.5%	44.4, 100	93.3%	76, 100	100%	85.7, 100
	Follow-up Diary	46.2	18.6, 77.1	1.79%	0, 3.88	87.5%	63.3, 97.0	92.1%	74.1, 100	92.1%	76.6, 100
		M	IQR	M	IQR	M	IQR	M	IQR	M	IQR
		*n* = 7		*n* = 4		*n* = 4		*n* = 11		*n* = 105	
**Withdrawn**	Follow-up Survey Withdrawn	14.3%	0, 25.9	0%	0, 6.25	35.7%	13.5, 62.3	10%	0, 39.2	15.4%	0, 46.2
	Follow-up Diary	8.33%	2.08, 65.2	1.79%	0, 3.88	87.5%	63.3, 97.0	92.1%	74.1, 100	92.1%	76.6, 100

Note: Participants in the existing Optimise groups were in the cohort for much longer, as recruitment for these groups began in September 2020 [See Figure 1]. Median and Interquartile range are reported.

## Data Availability

Data reports relevant to this paper can be found on the Optimise study website, https://www.burnet.edu.au/projects/459_the_optimise_study_optimising_isolation_quarantine_and_distancing_for_covid_19 (accessed on 20 November 2022).
